# Supplementary material for: Heat adaptation of phage T7 under an extended genetic code
Source: Virus Evol. 2021 Dec 1;7(2):veab100. doi: 10.1093/ve/veab100 (PMC8923235; doi:10.1093/ve/veab100)
Supplement: veab100_Supp [file veab100_supp.zip › Sequencing_Analysis.zip!/Sequencing_Analysis/S2/evidence/RA_21.html]

BRESEQ :: Evidence


| Read alignment evidence... | | | | | | | | | | | |
| --- | --- | --- | --- | --- | --- | --- | --- | --- | --- | --- | --- |
|  | seq id | position | | ref | new | freq | score (cons/poly) | reads | annotation | genes | product |
| \* | NC\_001604.1 | 24,238 | 0 | A | G | 28.8% | 6279.6 / inf | 5215 | intergenic (–/–) | *–/–* | –/– |
| Reads supporting (aligned to +/- strand):  **ref** base A (1879/1806);  **new** base G (755/735);  **total** (2665/2550) | | | | | | | | | | | |
| Fisher's exact test for biased strand distribution *p*-value = 8.54e-01 | | | | | | | | | | | |
| Kolmogorov-Smirnov test that lower quality scores support variant *p*-value = 1.00e+00 | | | | | | | | | | | |

Only 100 of 5269 total aligned reads displayed.  

`GTACGCAATGGGCCACGGTGGTCTTCGCCCAGAAGCTGCTGGTGCAGTGGTTTTCAAAGTGGAGTAATGCTGGGGGTGGCCTCAACGGTCGCTGCTAGTCCCGAAGAGGCGAGTGTTACTTCAACAGAAGAAACCTTAACGCCAGCACAGGAGGCCGCACGCACCCGCGCTGCTAACAAAGCCCGAAAGGAAGCTGAGTTGGCTGCTGCCACCGCTGAGCAATAACTAGCATAACCCCTTGGGGCCTCTAAACGGGTCTTGAGGGGTTTTTTGCTGAAAGGAGGAACTATATGCGCTCATACGATATGAACGTTGAGACTGCCGCTGAGTTATCAGCTGTGAACGACATTCTGGCGTCTATCGGTGAACCTCCGGTATCAACGCTGGAAGGTGACGCTAACGCAGATGCAGCGAACGCTCGGCGTATTCTCAACAAGATTAACCGACAGATTCAATCTCGTGGATGGACGTTCAACATTGAGGAAGGCATAACGCTACTACCTGATGTTTACTCCAACCTGATTGTATACAGTGACGACTATTTATCCCTAATGTCTACTTCCGGTCAATCCATCTACGTTAACCGAGGTGGCTATGTGTA  >  NC_001604.1/23938‑24538`  

                                                                                                                                                                                                                                                                                                            `|`                                                                                                                                                                                                                                                                                                              
          `tcggagatgggtataagagacagaGCTGCTGGTGCAGGGGTTTTCAAAGTGGAGTAATGCTGGGGGTGGCCTCAACGGTCGCTGCTAGTCCCGAAGAGGCGAGTGTTACTTCAACAGAAGAAACCTTAACGCCAGCACAGGAGGCCGCACGCACCCGCGCTGCTAACAAAGCCCGAAAGGAAGCTGAGTTGGCTGCTGCCACCGCTGAGCAATAACTAGCATAACCCCTTGGGGCCTCTAAACGGGTCTTGAGGGGTTTTTTGCTGAAAGGAGGAACTATATGCGCTCATGCGATATGAAc                                                                                                                                                                                                                                                                                                    <  1:72537/278‑1 (MQ=255)`  
          `ggCCACGGTGGTCTTCGCCCAGAAGCTGCTGGTGCAGTGGTTTTCAAAGTGGAGTAATGCTGGGGGTGGCCTCAACGGTCGCTGCTAGTCCCGAAGAGGCGAGTGTTACTTCAACAGAAGAAACCTTAACGCCAGCACAGGAGGCCGCACGCACCCGCGCTGCTAACAAAGCCCGAAAGGAAGCTGAGTTGGCTGCTGCCACCGCTGAGCAATAACTAGCATAACCCCTT‑GGGCCTCTAAACGGGTCTTGAGGGGTTTTTTGCTGAAAGGAGGAACTATATTCGCTCATACGATAAGAACg                                                                                                                                                                                                                                                                                                   >  1:286397/1‑301 (MQ=255)`  
           `gCCACGGTGGTCTTCGCCCAGAAGCTGCTGGTGCAGTGGTTTTCAAAGTGGAGTAATGCTGGGGGTGGCCTCAACGGTCGCTGCTAGTCCCGAAGAGGCGAGTGTTACTTCAACAGAAGAAACCTTAACGCCAGCACAGGAGGCCGCACGCACCCGCGCTGCTAACAAAGCCCGAAAGGAAGCTGAGTTGGCTGCTGCCACCGCTGAGCAATAACTAGCATAACCCCTTGGGGCCTCTAAACGGGTCTTGAGGGGTTTTTTGCTGAAAGGAGGAACTATATGCGCTCATACGATATGAACg                                                                                                                                                                                                                                                                                                   <  1:642425/301‑1 (MQ=255)`  
           `acagcGGTGGTCTTCGCCCAGAAGCTGCTGGTGCAGTGGTTTTCAAAGTGGAGTAATGCTGGGGGTGGCCTCAACGGTCGCTGCTAGTCCCGAAGAGGCGAGTGTTACTTCAACAGAAGAAACCTTAACGCCAGCACAGGAGGCCGCACGCACCCGCGCTGCTAACAAAGCCCGAAAGGAAGCTGAGTTGGCTGCTGCCACCGCTGAGCAATAACTAGCATAACCCCTTGGGGCCTCTAAACGGGTCTTGAGGGGTTTTTTGCTGAAAGGAGGAACTATATGCGCTCATACGATATGAACg                                                                                                                                                                                                                                                                                                   <  1:674207/297‑1 (MQ=255)`  
                 `gtggtCTTCGCCCAGAAGCTGCTGGTGCAGTGGTTTTCAAAGTGGAGTAATGCTGGGGGTGGCCTCAACGGTCGCTGCTAGTCCCGAAGAGGCGAGTGTTACTTCAACAGAAGATACCTTAACGCCAGCACAGGAGGCCGCACGCACCCGCGCTGCTAACAAAGCCCGAAAGGAAGCTGAGTTGGCTGCTGCCACCGCTGAGCAATAACTAGCATAACCCCTTGGGGCCTCTAAACGGGTCTTGAGGGGTTTTTTGCTGAAAGGAGGAACTTTATGCGCTCATACGATATGAGCGTTgaga                                                                                                                                                                                                                                                                                             >  1:405276/1‑301 (MQ=255)`  
                     `tCTTCGCCCAGAAGCTGCTGGTGCAGTGGTTTTCAAAGTGGAGTAATGCTGGGGGTGGCCTCAACGTTCGCTGCTAGTCCCGAAGAGGCTAGTGTTACTTCAACAGAAGAAACCTTTACTCCAGCACATCATGCCGCACGCTCCCTCTCTGCTAACACCTCCCGCAAGGAAGCTGAGTTGGCTCCTGCCACCGCTGAGCAATAACTAGCATAACCCCTTGGGTCCTCTAATCGGTCCTTTAGGGTTTTTTTGCTTTACGGATGTACTTTATTCCCTCATACGttactttttcttatactct                                                                                                                                                                                                                                                                                         >  1:456514/1‑285 (MQ=255)`  
                          `gacaggAAGCTGCTGGTGCAGTGGTTTTCAAAGTGGAGTAATGCTGGGGGTGGCCTCAACGGTCGCTGCTAGTCCCGAAGAGGCGAGTGTTACTTCAACAGAAGAAACCTTAACGCCAGCACAGGAGGCCGCACGCACCCGCGCTGCTAACAAAGCCCGAAAGGAAGCTGAGTTGGCTGCTGCCACCGCTGAGCAATAACTAGCATAACCCCTTGGGGCCTCTAAACGGGTCTTGAGGGGTTTTTTGCTGAAAGGAGGAACTATATGCGCTCATGCGATATGAACGTTGAGACTGCCGCTg                                                                                                                                                                                                                                                                                    <  1:143108/296‑1 (MQ=255)`  
                                         `gagatgtgtaaaagagacgggAGTAATGCTGGGGGTGGCCTCAACGGTCGCTGCTAGTCCCGAAGAGGCGAGTGTTACCTCAACAAAAGAAACCATAACGCCAGCACAGGGGGCCGCACGCACCCGCGCTGCTAACAAAGCCCGAAAGGACGCTGAGTTGGCTGCTGCCACCGCTGAGCAATAACTAGCATAACCCCTTGGGCCCTCTAAACGGGTCTTGAGGGG‑‑TTATGCTGAAAGGAGGAACTATATGCGCTCATGCGATATGAACGTTGAGACTGCCGCTGAGTTATCAGCTGTGAAc                                                                                                                                                                                                                                                                   <  1:575610/282‑1 (MQ=255)`  
                                           `gCAGTGGTTTTCAAAGTGGAGTAATGCTGGGGGTGGCCTCAACGGTCGCTGCTAGTCCCGAAGAGGCGAGTGTTACTTCAACAGAAGAAACCTTAACGCCAGCACAGGAGGCCGCACGCACCCGCGCTGCTAACAAAGCCCGAAAGGAAGCTGAGTTGGCTGCTGCCACCGCTGAGCAATAACTAGCATAACCCCTTGGGGCCTCTAAACGGGTCTTGAGGGGTTTTTTGCTGAAAGGAGGAACTATATGCGCTCATACGATATGAACGTTGAGACTGCCGCTGAGTTATCAGCTGTGAAc                                                                                                                                                                                                                                                                   <  1:361177/301‑1 (MQ=255)`  
                                              `gtgtTTTTCAAAGTGGAGTAATGCTGGGAGTGGCCTCAACGGTCGCTGCTAGTCCCGAAGAGGCGAGTGTTACTTCAACAGAAGAAACCTTAACGCCAGCACAGGAGGCCGCACGCACCCGCGCTGCTAACAAAGCCCGAAAGGAAGCTGAGTTGGCTGCTGCCACCGCTGAGCAATAACTAGCATAACCCCTTGGGGCCTCTAAACGGGTCTTGAGGGGTTTTTTGCTGAAAGGAGGAACTATATGCGCTCATACGATATGAACGTTGAGACTGCCGCTGAGTTATCAGCTGTGAACGAc                                                                                                                                                                                                                                                                >  1:717920/1‑301 (MQ=255)`  
                                              `gTGGTTTTCAAAGTGGAGTGATGCTGGGGGTGGCCTCAACGGTCGCTGCTAGTCCCGACGAGGCGAGTGTTACTTCAACATAAGAAACCTTAACGCCAGCACAGGAGGCCGCACGCACCCGCGCTGCTAACAAAGCCCGAAAGGAAGCTGAGTTGGCTGCTGCCACCGCTGAGCAATAACTAGCATAACCCCTTGGGGCCTCTAAACGGGTCTTGAGGGGTTTTTTGCTGAAAGGAGGAACTATATGCGCTCATACGATATGAACGTTGAGACTGCCGCTGAGTTATCAGCTGTGAACGAc                                                                                                                                                                                                                                                                <  1:150669/301‑1 (MQ=255)`  
                                              `gTGGTTTTCAAAGTGGAGTAATGCTGGGGGTGGCCTCAACGGTCTCTGCTAGTCCCGAAGAGGCGAGTGTTACTTCAACAGAAGAAACCTTAACGCCAGCACAGGAGGCCGCACGCACCCGCGCTGCTAACAAAGCCCGAAAGGAAGCTGAGTTGGCTGCTGCCACCGCTGAGCAATAACTAGCATAACCCCTTGGGGCCTCTAAACGGGTCTTGAGGGGTTTTTTGCTGAAAGGAGGAACTATATGCGCTCATGCGATATGAACGTTGAGACTGCCGCTGAGTTATCAGCTGTGAACGAc                                                                                                                                                                                                                                                                >  1:559887/1‑301 (MQ=255)`  
                                              `gTGGTTTTCAAAGTGGAGTAATGCTGGGGGTGGCCTCAACGGTCGCTGCTAGTCCCGAAGAGGCGAGTGTTACTTCAACAGAAGAAACCTTAACGCCAGCACAGGAGGCCGCACGCACCCGCGCTGCTAACAAAGCCCGAAAGGAAGCTGAGTTGGCTGCTGCCACCGCTGAGCAATAACTAGCATAACCCCTTGGGGCCTCTAAACGGGTCTTGAGGGGTTTTTTGCTGAAAGGAGGAACTATATGCGCTCATGCGATATGAACGTTGAGCCTGCCGCTGAGTTATCAGCTGTGAACgcc                                                                                                                                                                                                                                                                >  1:263709/1‑299 (MQ=255)`  
                                                 `gTTTTCAAAGTGGAGTAATGCTGGGGGTGGCCTCAACGGTCGCTGCTAGTCCCGAAGAGGCGAGTGTTACTTCAACAGAAGAAACCTTAACGCCAGCACAGGAGGCCGCACGCACCCGCGCTGCTAACAAAGCCCGAAAGGAAGCTCAGTTGGCTGCTGCCACCGCTGCGCAATAACTAGCATAACCCCTTGGGGCCTCTAAACGGGTCTTGAGGGGTTTTTTGCTGAAAGGAGGAACTATATGCGCTCATACGATATGAACGTTGCGACTGCCGCTGAGTTATCAGCTGTGAACGACAtt                                                                                                                                                                                                                                                             >  1:248940/1‑301 (MQ=255)`  
                                                     `tCAAAGTGGAGTAATGCTGGGGGGGGCCTCAACGGTCGTTGCTAGTCCCGAAGAGGCGAGTGTTACTTCAACAGAAGAAACCTTAACGCCAGCACAGGAGGCCGCACGCACCCGCGCTGCTAACAAAGCCCGAAAGGAAGCTGAGTTGACTGCTGCCACCGCTGAGCAATAACTAGCATAACCCCTTGGGGCCTCTAAACGGGTCTTGAGGGGTTTTTTGCTGAAAGGAGGAACTATATGCGCTCATGCGATATGAACGTTGAGACTGCCGCTGAGTTATCAGCTGTGAACGACATTCTgg                                                                                                                                                                                                                                                         <  1:352161/301‑1 (MQ=255)`  
                                                        `aaGTGGAGTAATGCTGGGGGTGGCCTCAACGGTCGCTGCTAGTCCCGAAGAGGCGAGTGTTACTTCAACAGAAGAAACCTTAACGCCAGCACAGGAGGCCGCACGCACCCGCGCTGCTAACAAAGCCCGAAAGGAAGCTGAGTTGGCTGCTGCCACCGCTGAGCAATAACTAGCATAACCCCTTGGGGCCTCTAAACGGGTCTTGAGGGGTTTTTTGCTGAAAGGAGAAACTATATGCGATCATACGATATGAACGTTGAGACTGCCGCTGAGTTATCAGCTGTGAACGACATTCTGGCgg                                                                                                                                                                                                                                                      >  1:701692/1‑300 (MQ=255)`  
                                                         `aGTGGAGTAATGCTGGGGGTGGCCTCAACGGTCGCTGCTAGTCCCGAAGAGGCGAGTGTTACTTCAACAGAAGAAACCTTAACGCCAGCACAGGAGGCCGCACGCACCCGCGCTGCTAACAAAGCCCGAAAGGAAGCTGAGTTGGCTGCTGCCACCGCTGAGCAATAACTAGCATAACCCCTTGGGGCCTCTAAACGGGTCTTGAGGGGTTTTTTGCTGAAAGGAGGAACTATATGCGCTCATACGATATGAACGTTGAGACTGCCGCTGAGTTATCAGCTGTGAACGACATTCTGGCGTc                                                                                                                                                                                                                                                     <  1:127584/301‑1 (MQ=255)`  
                                                          `gTGGAGTAATGCTGGGGGTGGCATCAACGGTCGCTGCTAGTCCCGAAGAGGCGAGTGTTACTTCAACAGAAGAAACCTTAACGCCAGCACAGGAGGCCGCACGCACCCGCGCTGCTAACAAAGCCCGAAAGGAAGCTGAGTTGGCTGCTGCCACCGCTGAGCAATAACTAGCATAACCCCTTGGGGCCTCTAAACGGGTCTTGAGGGGTTTTTTGCTGAAAGGAGGAACTATATGCGCTCATACGATATGAACGTTGAGACTGCCGCTGAGTTATCactgtctcttatacacatctccgag                                                                                                                                                                                                                                                    >  1:782106/1‑277 (MQ=255)`  
                                                               `acggtGCTGGGGGTGGCCCTAACGGTCGCTGCTAGTCCCGAAGAGGCGAGTGTTAATTCAACAGAAGAAACCTTAACGCCAGCACAGGAAGCCGCACGAACCAGCGCTGCTAACAAAGCCCGAAAGGAAGCTGAGTTGGCTGCTGACACCGCTGAGCAATAACTAGCATAACCCCTTGGGGCCTCTAAACGGGTCTTGAGGGGTTTTTTGCTGAAAGGAGGAACTATATGCGCTCATACGATATGAACGTTGAGACTGCCGATGAGTTATCAGCTGTGAACGACATTCTGGCGTCTATCgg                                                                                                                                                                                                                                               <  1:749474/297‑1 (MQ=255)`  
                                                                  `aTGCTGGGGGTGGCCTCAACGGTCGCTGCTAGTCCCGAAGAGGCGAGTGTTACTTCAACAGAAGAAACCTTAACGCCAGCACAGGAGGCCGAACGCACCCGCGCTGCTAACAAAGCCCGAAAGGAAGCTGAGTTGGCTGCTGCCACCGCTGAGCAATAACTAGAATAACCCCTTGGGGCCTCTAAACGGGTCTTCAGTGGTTTTTTGCTGAAAGGAGGCACTATATGCGCTCATGCGATATGAACGTTGAGACTGCCGCTGAATTATCAGCTGTAACCTACATTCTGGCGTCTCTCTGTGa                                                                                                                                                                                                                                            >  1:479595/1‑301 (MQ=255)`  
                                                                   `tGCTGGGGGTGGCCTCAACGGTCGCTGCTAGTCCCGAAGAGGCGAGTGTTACTTCAACAGAAGAAACCTTAACGCCAGCACAGGAGGCCGCACGCACCCGCGCTGCTAACAAAGCCCGAAAGGAAGCTGAGTTGGCTGCTGCCACCGCTGAGCAATAACTAGCATAACCCCTTGGGGCCTCTAAACGGGTCTTGAGGGGTTTTTTGCTGAAAGGAGGAACTATATGCGCTCATACGATATGAACGTTGAGACTGCCGCTGAGTTATCAGCTGTGAACGAcctgtctcttatacacatctcc                                                                                                                                                                                                                                           >  1:226868/1‑280 (MQ=255)`  
                                                                   `tGCTGGGGGTGGCCTCAACGGTCGCTGCTAGTCCCGAAGAGGCGAGTGTTACTTCAACAGAAGAAACCTTAACGCCAGCACAGGAGGCCGCACGCACCCGCGCTGCTAACAAAGCCCGAAAGGAAGCTGAGTTGGCTGCTGCCACCGCTGAGCAATAACTAGCATAACCCCTTGGGGCCTCTAAACGGGTCTTGAGGGGTTTTTTGCTGAAAGGAGGAACTATATGCGCTCATACGATATGAACGTTGAGACTGCCGCTGAGTTATCAGCTGTGAACGACATTCTGGctgtctcttataca                                                                                                                                                                                                                                           >  1:530758/1‑288 (MQ=255)`  
                                                                    `gCTGGGGGTGGCCTCAACGGTCGCTGCTAGTCCCGAAGAGGCGAGTGTTACTTCAATAGAAGAAACCTTAACGCCAGCACAGGAGGCCGCACGCACCCGCGCTGCTAACAAAGCCCGAAAGGAAGCTGAGTTGGCTGCTGCCACCGCTGAGCAATAACTAGCATAACCCCGTGGGGCCTCTAAACGGGTCTTGAGGGGTTTTTTGCTGAAAGGAGGAACTATATGCGCTCATACGATATGAACGTTGAGACTGCCGCTGAGTTATCAGCTGTGAACGACATTCTGGCGTCTATCGGTGAAc                                                                                                                                                                                                                                          <  1:106666/301‑1 (MQ=255)`  
                                                                    `gCTGGGGGTGGCCTCAACGGTCGCTGCTAGTCCCGAAGAGGCGAGTGTTACTTCAACAGAAGAAACCTTAACGCCAGCACAGGAGGCCGCACGCACCCGCGCTGCTAACAAAGCCCGAAAGGAAGCTGAGTTGGCTGCTGCCACCGCTGAGCAATAACTAGCATAACCCCTTGGGGCCTCTAAACGGGTCTTGAGGGGTTTTTTGCTGAAAGGAGGAACTATATGCGCTCATACGATATGAACGTTGAGACTGCCGCTGAGTTATCAGCTGTGAAAGACATTCTGGCGTCTATCGGTGAAc                                                                                                                                                                                                                                          >  1:774364/1‑301 (MQ=255)`  
                                                                       `gggggTGGCCTCAACGGTCGCTGCTAGTCCCGAAGAGGCGAGTGTTACTTCAACAGAAGAAACCTTAACGCCAGCACAGGAGGCCGCACGCACCCGCGCTGCTAACAAAGCCCGAAAGGAAGCTGAGTTGGCTGCTGCCACCGCTGAGCAATAACTAGCATAACCCCTTGGGGCCTCTAAACGGGTCTTGAGGGGTTTTTTGCTGAAAGGAGGAACTATATGCGCTCATGCGATATGAACGTTGAGACTTCCGCTGAGTTATCAGCTGTGAACGACATTCTGGCGTCTATCGGTGAACCTc                                                                                                                                                                                                                                       >  1:464274/1‑301 (MQ=255)`  
                                                                         `gggTGGCCTCAACGGTCGCTGCTAGTCCCGAAGAGGCGAGTGTTACTTCAACAGAAGAAACCTTAACGCCAGCACAGGAGGCCGCACGCACCCGCGCTGCTAACAAAGCCCGAAAGGAAGCTGAGTTGGCTGCTGCCACCGCTGAGCAATAACTAGCATAACCCCTTGGGGCCTCTAAACGGGTCTTGAGGGGTTTTTTGCTGAAAGGAGGAACTATATGCGCTCATACGATATGAACGTTGAGACTGCCGCTGAGTTATCAGCTGTGAACGACATTCTGGctgtctcttatacacatctc                                                                                                                                                                                                                                     >  1:487105/1‑282 (MQ=255)`  
                                                                             `ggCCTCAACGGTCGCTGCTAGTCCCGAAGAGGCGAGTGTTACGTCAACAGAAGAAACCTTAACGCCAGCACAGGAGGCAGAACGCACCCGCGCTGCTAACAAAGCCCGAAAGGAAGCTGAGTTGGCTGCTGCCACCGCTGAGCAATAACTAGCATAACCCCTTGGGGCCTCTAAACGGGTCTTGAGGGGTTTTTTGCTGAAAGGAGGAACTATATGCGCTCATACGATATGAACGTTGAGACTGCCGCTGAGTTATCAGCTGTGAACGACATTCTGGCGTCTATCGGTGAACCTCCGGTAt                                                                                                                                                                                                                                 <  1:322484/301‑1 (MQ=255)`  
                                                                             `gagatgtgtataagagacaggTCCCGAAGAGGCGAGTGTTACTTCAACAGAAGAAACCTTAACGCCAGCACAGGAGGCCGCACGCACCCGCGCTGCTAACAAAGCCCGAAAGGAAGCTGAGTTGGCTGCTGCCACCGCTGAGCAATAACTAGCATAACCCCTTGGGGCCTCTAAACGGGTCTTGAGGGGTTTTTTGCTGAAAGGAGGAACTATATGCGCTCATACGATATGAACGTTGAGACTGCCGCTGAGTTATCAGCTGTGAACGACATTCTGGCGTCTATCGGTGAACCTCCAGTAt                                                                                                                                                                                                                                 <  1:741803/281‑1 (MQ=255)`  
                                                                                  `cAACGGTCGCTGCTAGTCCCGAAGAGGCGAGTGTTACTTCAACAGAAGAAACCTTAACGCCAGCACAGGAGGCCGCACGCACCCGCGCTGCTAACAAAGCCCGAAAGGAAGCTGAGTTGGCTGCTGCCACCGCTGAGCAATAACTAGCATAACCCCTTGGGGCCTCTAAACGGGTCTTGAGGGGTTTTTTGCTGAAAGTAGGAACTATATGCGCTCATACGATATGAACGTTGAGACTGCCGCTGAGTTATCAGCTGTGAACGACATTCTGGCGTCTATCGGTGAACCTCCGGTATCAACg                                                                                                                                                                                                                            >  1:821684/1‑301 (MQ=255)`  
                                                                                   `aaCGGTCGCTGCTAGTCCCGAAGAGGCGAGTGTTACTTCAACAGAAGAAACCTTAACGCCAGCACAGGAGGCCGCACGCACCCGCGCTGCTAACAAAGCCCGAAAGGAAGCTGAGTTGGCTGCTGCCACCGCTGAGCAATAACTAGCATAACCCCTTGGGGCCTCTAAACGGGTCTTGAGGGGTTTTTTGCTGAAAGGAGGAACTATATGCGCTCATACGATATGAACGTTGAGACTGCCGCTGAGTTATCAGCTGTGAACGACATTCTGGCGTCTATCGGTGAACCTCCGGTATCAACGc                                                                                                                                                                                                                           <  1:797051/301‑1 (MQ=255)`  
                                                                                          `gctgctAGTCCCGAAGAGGCGAGTGTTACTTCAACAGAAGAAACCTTAACGCCAGCACAGGAGGCCGCACGCACCCGCGCTGCTAACAAAGCCCGAAAGGAAGCTGAGTTGGCTGCTGCCACCGCTGAGCAATAACTAGCATAACCCCTTGGGGCCTCTAAACGGGTCTTGAGGGGTTTTTTGCTGAAAGGAGGAACTATATGCGCTCATGCGATATGAACGTTGAGACTGCCGCTGAGTTATCAGCTGTGAACGACATTCTGGCGTCTATCGGTGAACCTCCGGTATCAACGCTGGAAgg                                                                                                                                                                                                                    <  1:158193/301‑1 (MQ=255)`  
                                                                                          `gagacaggTCCCGAAGAGGCGAGTGTTACTTCAACAGAAGAAACCTTAACGCCAGCACAGGAGGCCGCACGCACCCGCGCTGCTAACAAAGCCCGAAAGGAAGCTGAGTTGGCTGCTGCCACCGCTGAGCAATAACTAGCATAACCCCTTGGGGCCTCTAAACGGGTCTTGAGGGGTTTTTTGCTGAAAGGAGGAACTATATGCGCTCATGCGATATGAACGTTGAGACTGCCGCTGAGTTATCAGCTGTGAACGACATTCTGGCGTCTATCGGTGAACCTCCGGTATCAACGCTGGAAgg                                                                                                                                                                                                                    <  1:538747/294‑1 (MQ=255)`  
                                                                                           `ctgctAGTCCCGAAGAGGCGAGTGTTACTTCAACAGAAGAAACCTTAACGCCAGCACAGGAGGCCGCACGCACCCGCGCTGCTAACAAAGCCCGAAAGGAAGCTGAGTTGGCTGATGCCACCGCTGAGCAATAACTAGCATAACCCCTTGGGGCCTCTAAACGGGTCTTGAGGGGTTTTTTGCTGAAAGGAGGAACTATATGCGCTCATACGATATGAACGTTGAGACTGCCGCTGAGTTATCAGCTGTGAACGACATTCTGGCGTCTATCGGTGAACCTCCGGTATCAACGCTGGAAggg                                                                                                                                                                                                                   >  1:688921/1‑300 (MQ=255)`  
                                                                                                      `ttggtataagagacagcTTCAACAGAAGAAACCTTAACGCCAGCACAGGAGGCCGAACGCACCCGCGCTGCTAACAAAGCCCGAAAGGAAGCTGAGTTGGCTGCTGCCACCGCTGAGCAATAACTAGCATAACCCCTTGGGGCCTCTAAACGGGTCTTGAGGGGTTTTTTGCTGAAAGGAGGAACTATATGCGCTCATGCGATATGAACGTTGAGACTGCCGCTGAGTTATCAGCTGTGAACGACATTCTGGCGTCTATCGGTGAACCTCCGGTATCAACGCTGGAAGGTGACGCTAACGc                                                                                                                                                                                                        <  1:43980/285‑1 (MQ=255)`  
                                                                                                       `aaGAGGCGAGTGTTACTTCAACAGAAGAAACCTTAACGCCAGCACAGGAGGCCGAACGCACCCGCGCTGCTAACAAAGCCCGAAAGGAAGCTGAGTTGGCTGCTGCCACCGCTGAGCAATAACTAGCATAACCCCTTGGGGCCTCTAAACGGGTCTTGAGGGGTTTTTTGCTGAAAGGAGGAACTATATGCGCTCATACGAAATGAACGTTGAGACTGCCGCTGAGTTATCAGCTGTGAAGGACATTCTGGCGTCTATCGGTGAACCTCAGGTCTCAACGATGGAAGGTGACGCTAACGCa                                                                                                                                                                                                       >  1:635236/1‑301 (MQ=255)`  
                                                                                                           `ggCGAGTGTTACTTCAACAGAAGAAACCTTAACGCCAGCACAGGAGGCCGCACGCACCCGCGCTGCTAACAAAGCCCGAAAGGAAGCTGAGTTGGCTGCTGCCACCGCTGAGCAATAACTAGCATAACCCCTTGGGGCCTCTAAACGGGTCTTGAGGGGTTTTTTGCTGAAAGGAGGAACTATATGCGCTCATACGATATGAACGTTGAGCCTGCCGCTGAGTTATCAGCTGTGAACGCCCTTCTGGCGTCTATCGGTGAACCTCCGGTATCAACGCTGGAAGGTGACGCTAACGCAGATg                                                                                                                                                                                                   >  1:614233/1‑301 (MQ=255)`  
                                                                                                                `gtgtTACTTCAACAGAAGAAACCTTAACGCCAGCACAGGAGGCCGCACGCACCCGCTCTGCTAACAAAGCCCGAAAGGAAGCTGAGTTGGCTGCTGCCACCGCTGAGCAATAACTAGCATAACCCCTTGGGGCCTCTAAACGGGTCTTGAGGGGTTTTTTGCTGAAAGGAGGAACTATATGCGCTCATACGATATGAACGTTGAGACTGCCGCTGAGTTATCAGCTGTGAACGACATTCTGGCGTCTATCGGTGAACCTCCGGTATCAACGCTGGAAGGTGACGCTAACGCAGATGCAGCg                                                                                                                                                                                              >  1:667744/1‑301 (MQ=255)`  
                                                                                                                    `tACTTCAACAGAAGAAACCTTAACGCCAGCACAGGAGGCCGCACGCACCCGCGCTGCTAACAAAGCCCGAAAGGAAGCTGAGTTGGCTGCTGCCACCGCTGAGCAATAACTAGCATAACCCCTTGGGGCCTCTAAACGGGTCTTGAGGGGTTTTTTGCTGAAAGGAGGAACTATAGGCGCTCATACGATATGAACGTTGAGACTGCCGCTGAGCTATCAGCTTTGAACGACATTCTGGCGTCTATCGGTGAACCTCCGGTATCAACGCTGGAAGGTGACGCTAACGCAGATGCAGCGAACg                                                                                                                                                                                          >  1:788771/1‑301 (MQ=255)`  
                                                                                                                         `cAACAGAAGAAACCTTAACGCCAGCACAGGAGGCCGCACGCACCCGCGCTGCTAACAAAGCCCGAAAGGAAGCTGAGTTGGCTGCTGCCACCGCTGAGCAATAACTAGCATAACCCCTTGGGGCCTCTAAACGGGTCTTGAGGGGTTTTTTGCTGACAGGCGGAACTATATGCGCTCATACGATATGAACGTTGAGACTGCCGCTGAGTTATCAGCTGTGAACGACATTCTGGCGTCTATCGGTGAACCTCCGGTATCAACGCTGGAAGGTGACGCTAACGCAGATGCAGCGAACGCTCgg                                                                                                                                                                                     >  1:545442/1‑301 (MQ=255)`  
                                                                                                                              `gaagaaACCTTAACGCCAGCACAGGAGGCCGCACGCACCCGCGCTGCTAACAAAGCCCGAAAGGAAGCTGAGTTGGCTGCTGCCACCGCTGAGCAATAACTAGCATAACCCCTTGGGGCCTCTAAACGGGTCTTGAGGGGTTTTTTGCTGAAAGGAGGAACTATATGCGCTCATGCGATATGAACGTTGAGACTGCCGCTGAGTTATCAGCTGTGAACGACATTATGGCGTCTATCGGTGAACCTCCGGTATCAACGCTGGAAGGTGACGCTAACGCAGATGCAGCGAACGCTCGGCGTAt                                                                                                                                                                                >  1:272140/1‑301 (MQ=255)`  
                                                                                                                              `gaagaaACCTTAACGCCAGCACAGGAGGCCGCACGCACCCGCGCTGCTAACAAAGCCCGAAAGGAAGCTGAGTTGGCTGCTGCCACCGCTGAGCAATAACTAGCATAACCCCTTGGGGCCTCTAAACGGGTCTTGAGGGGTTTTTTGCTGAAAGGAGGAACTATATGCGCTCATACGATATGAACGTTGAGACTGCCGCTGAGTTATCAGCTGTGAACGACATTCTGGCGTCTATCGGTGAACCTCCGGTATCAACGCTGGAAGGTGACGCTAACGAAGATGCAGCGAATGCTCGGCGTAt                                                                                                                                                                                >  1:256269/1‑301 (MQ=255)`  
                                                                                                                                `agaaAACCTAACGCCCGGACAGGAGGCCGCACGCACCCGCGCTGCTAACAAAGCCCGAAAGGAAGCTGAGTTGGCTGCTGCCACCGCTGAGCAATAACTAGCATAACCCCTTGGGGCCTCTAAACGGGTCTTGAGGGGTTTTTTGCTGAAAGGAGGAACTATATGCGCTCATGCGATATGAACGTTGAGACTGCCGCTGAGTTATCAGCTGTGAACGACATTCTGGCGTCTATCGGTGAACCTCCGGTATCAACGCTGGAAGGTGACGCTAACGCAGATGCAGCGAACGCTCGGCGTATtc                                                                                                                                                                              <  1:432519/301‑1 (MQ=255)`  
                                                                                                                                     `ccTTAACGCCAGCACAGGAGGCCGCACGCACCCGCGCTGCTAACAAAGCCCGAAAGGAAGCTGAGTTGGCTGCTGCCACCGCTGAGCAATAACTAGCATAACCCCTTGGGGCCTCTAAACGGGTCTTGAGGGGTTTTTTGCTGAAAGGAGGAACTATATGCGCTCATACGATATGAACGTTGAGACTGCCGCTGAGTTATCAGCTGTGAACGACATTCTGGCGTCTATCGGTGAACCTCCGGTATCAACGCTGGAAGGTGACGCTAACGCAGATGCAGCGAACGCTCGGCGTATTCTcaaa                                                                                                                                                                         >  1:734679/1‑300 (MQ=255)`  
                                                                                                                                      `cTTAACGCCAGCACAGGAGGCCGCACGCACCCGCGCTGCTAACAAAGCCCGAAAGGAAGCTGAGTTGGCTGCTGCCACTGCTGAGCAATAACTAGCATAACCCCTTGGGGCCTCTAAACGGGTCTTGAGGGGTTTTTTGCTGAAAGGAGGAACTATATGCGCTCATACGATATGAACGTTGAGACTGCCGCTGAGTTATCAGCTGTGAACGACATTCTGGCGTCTATCGGTGAACCTCCGGTATCAACGCTGGAAGGTGACGCTAACGCAGATGCAGCGAACGCTCGGCGTATTCTcaaca                                                                                                                                                                        >  1:308874/1‑301 (MQ=255)`  
                                                                                                                                          `gcGCCAGCACAGGAGGCCGCACGCACCCGCGCTGCTAACAAAGCCCGAAAGGAAGCTGAGTTGGCGGCTGCCACCGCTGAGCAATAACTAGCATAACCCCTTGGGGCCTCTAAACGGGTCTTGAGGGGTTTTTTGCTGAAAGGAGGAACTATATGAGCTCATACGATATGAACGTTGAGACTGACGCTGAGTTATCAGCTGTGAACGACATTCTGGCGTCGATCGGTGAACCTCCGGTATCAACGCTGGAAGGTGACGCTAACGCAGATGCCGCGAACGCTCGGCGTATTCTCAACAAGAt                                                                                                                                                                    <  1:47170/300‑1 (MQ=255)`  
                                                                                                                                          `aCGCCAGCACAGGAGGCCGCACGCACCCGCGCTGCTAACAAAGCCCGAAAGGAAGCTGAGTTGGCTGCTGCCACCGCTGAGCAATAACTAGCATAACCCCTTGGGGCCTCTAAACGGGTCTTGAGGGGTTTTTTGCTGAAAGGAGGAACTATATGCGATCATACGATATGAACGTTGAGACTGCCGCTGAGTTATCAGCTGTGTACGACATTCTGGCGTCTATCGGTGAACCTCCGGTATCAACGCTGGAAGGTGACGCTAACGCAGATGCAGCGAACGCTCGGCGTATTCTCAACAAGAt                                                                                                                                                                    <  1:384197/301‑1 (MQ=255)`  
                                                                                                                                             `ccATCACAGGAGGCCGCACGCACCCGCGCTGCTAACAAAGCCCGAAAGGAAGCTGAGTTGGCTGCTGCCACCGCTGAGCAATAACTAGCATAACCCCTTGGGGCCTCTAAACGGGTCTTGAGGGGTTTTTTGCTGAAAGGAGGAACTATATGCGCTCATACGATATGAACGTTGAGACTGCCGCTGAGTTATCAGCTGTGAACGACATTCTGGCGTCTATCGGTGAACCTCCGGTATCAACGCTGGAAGGTGACGCTAACGCAGATGCAGCGAACGCTCGGCGTAtctgtctcttatacac                                                                                                                                                                 >  1:583858/1‑286 (MQ=255)`  
                                                                                                                                               `agaccatgaggCCGCACGCACCCGCGCTGCTAACAAAGCCCGAAAGGAAGCTGAGTTGGCTGCTGCCACCGCTGAGCAACAACTAGCATAACCCCTTGGGGCATCTAAACGGGTCTTGAGGGGTTTTTTGCTGAAAGGAGGAACTATATGCGCTCATGCGATATGAACGTTGAGACTGCCGCTGAGTTATCAGCTGTGAACGACATTCTGGCGTCTATCGGTGAACCTCCGGTATCAACGCTGGAAGGTGACGCTAACGCAGATGCAGCGAACGCTCGGCGTATTCTCAACAAGATTAAcc                                                                                                                                                               <  1:628407/294‑1 (MQ=255)`  
                                                                                                                                                `gCACAGGAGGCCGCACGCACCCGCGCTGCTAACAAAGCCCGAAAGGAAGCTGAGTTGGCTGCTGCCACCGCTGAGCAATAACTAGCATAACCCCTTGGGGCCTCTAAACGGGTCTTGAGTGGTTTTTTGCTGAAAGGAGGAACTATATGCGCTCATGCGATATGAACGTTGAGACTGCCGCTGAGTTATCAGCTGTGAACGACATTCTGGCGTCTATCGGTGAACCTCCGGTATCAACGCTGGAAGGTGACGCTAACGCAGATGCAGCGAACGCTCGGCGTATTCTCAACAAGATTAACCg                                                                                                                                                              >  1:191964/1‑301 (MQ=255)`  
                                                                                                                                                `gCACAGGAGGCCGCACGCACCCGCGCTGCTAACAAAGCCCGAAAGGAAGCTGAGTTGGCTGCTGCCACCGCTGAGCAATAACTAGCATAACCCCTTGGGGCCTCTAAACGGGTCTTGAGGGGTTTTTTGCTGAAAGGAGGAACTATATGCGCTCATGCGATATGAACGTTGAGACTGCCGCTGAGTTATCAGCTGTGAACGACATTCTAGCGTCTATCGGTGAACCTCCGGTATCAACGCTGGAAGGTGACGCTAACGCAGATGCAGCGAACGCTCGGCGTATTCTCAACAAGATTAAccc                                                                                                                                                              >  1:553422/1‑300 (MQ=255)`  
                                                                                                                                                  `acaGGAGGCCGCACGCACCCGCGCTGCTAACAAAGCCCGAAAGGAAGCTGAGTTGGCTGCTGCCACCGCTGAGCAATAACTAGCATAACCCCTTGGGGCCTCTAAACGGGTCTTGAGGGGTTTTTTGCTGAAAGGAGGAACTATATGAGCTCATACGATATGAACGTTGAGACTGCCGCTGAGTTACCAGCTGTGAACGACATTCTGGCGTCTATCGGTGAACCTCCGGTATCAACGCTGGAAGGTGACGCTAACGCAGATGCAGCGAACGCTCGGCGTATTCTCAACAAGATTAACCGAc                                                                                                                                                            <  1:119210/301‑1 (MQ=255)`  
                                                                                                                                                   `caGGAGGCCGCACGCACCCGCGCTGCTAACAAAGCCCGAAAGGAAGCTGAGTTGGCTGCTGCCACCGCTGAGCAATAACTAGCATAACCCCTTGGGGCCTCTAAACGGGTCTTGAGGGGTTTTTTGCTGAAAGGAGGAACTATATGCGCTCATGCGATATGAACGTTGAGACTGCCGCTGAGTTATCAGCTGTGAACGACATTCTGGCGTCTATCGGTGAACCTCCGGTATCAACGCTGGAAGGTGACGCTAACGCAGATGCAGCGAACGCTCGGCGTATTCTCAACAAGATTAACCGACa                                                                                                                                                           >  1:90662/1‑301 (MQ=255)`  
                                                                                                                                                            `gcccccacCAGGGCTGCAAACAAAGCCCGAAAGGAAGCTGAGTTGGCTGCTGCCTCCGCTGAGCAATAACTAGCAGAACCCCTTGGGGCCTCTAAACGGGTCTTGAGGGGTTTTTTGCTGAAAGGAGGAACTATATGCGCTCATGCGATATGAACGTTGAGACTGCCGCTGAGTTATCAGCTGTGAACGACATTCTGGCGTCTATCGGTGAACCTACGGTATCAACGCTGGAAGGTGACGCTAACGCAGATGCAGCGAACGCTCGGCGTATTCTCAACAAGATTAACCGACAGATTCAAtc                                                                                                                                                  <  1:814217/296‑1 (MQ=255)`  
                                                                                                                                                            `gacagccaccGCGCTGCTAACAAAGCCCGAAAGGAAGCTGAGTTGGCTGCTGCCACCGCTGAGCAATAACTAGCATAACCCCTTGGGGCCTCTAAACGGGTCTTGAGGGGTTTTTTGCTGAAAGGAGGAACTATATGCGCTCATACGATATGAACGTTGAGACTGCCGCTGAGTTATCAGCTGTGAACGACATTCTGGCGTCTATCGGTGAACCTCCGGTATCAACGCTGGAAGGTGACGCTAACGCAGATGCAGCGAACGCTCGGCGTATTCTCAACAAGATTAACCGACAGATTCAAtc                                                                                                                                                  <  1:200333/293‑1 (MQ=255)`  
                                                                                                                                                             `cggagatgtgtataagagaCAGAGCCCGAAAGGAAGCTGAGTTGGCTGCTGCCACCGCGGAGCAATAACTAGCATAACCCCTTGGGGCCTCTAAACGGGTCTTGAGGGGTTTTTTGCTGAAAGGAGGAACTATATGCGCTCATACGATATGAACGTTGAGACTGCCGCTGAGTTATCAGCTGTGAACGACATTCTGGCGTCTATCGGTGAACCTCCGGTATCAACGCTGGAAGGTGACGCTAACGCAGATGCAGCGAACGCTCGGCGTATTCTCAACAAGATTAACCGACAGATTCAAtct                                                                                                                                                 <  1:335828/283‑1 (MQ=255)`  
                                                                                                                                                             `aacgaacCCGCGCTGCTAACAAAGCCCGAAAGGAAGCTGAGTTGGCTGCTGCCAACGCTGAGCAATAACTAGCATAACCCCTTGGGGACTCTAAACGGGTCTTGAGGGGTTTTTTGCTGAAAGGAGGAACTATATGCGCTCATACGATATGAACGTTGAGACTGCCGCTGAGTTATCAGCTGTGAACGACATTCTGGCGTCTATCGGTGAACCTCCGGTATCAACGCTGGAAGGTGACGCTAACGCAGATGCAGCGAACGCTCGGCGTATTCTCAACAAGATTAACCGACAGATTCAAtct                                                                                                                                                 <  1:178546/300‑1 (MQ=255)`  
                                                                                                                                                                   `cccGCGCTGCTAACAAAGCCCGAAAGGAAGCTGAGTTGGCTGCTGCCACCGCTGAGCAATAACTAGCATAACCCCTTGGGGCCTCTAAACGGGTCTTGAGGGGTTTTTTGCTGAAAGGAGGAACTATATGCGCTCATGCGATATGAACGTTGAGACTGCCGCTGAGTTATCAGCTGTGAACGACATTCTGGCGTCTATCGGTGAACCTCCGGTATCAACGCTGGAAGGTGACGCTAACGCAGATGCAGCGAACGCTCGGCGTATTCTCAACAAGATTAAccctgtctcttatacacatctc                                                                                                                                           >  1:621845/1‑281 (MQ=255)`  
                                                                                                                                                                          `tgctAACAAAGCCCGAAAGGAAGCTGAGTT‑GCTGCTGCCACCGCTGAGCAATAACTAGCATAACCCCTTGGGGCCTCTAAACGGGTCTTGAGGGGTTTTTTGCTGAAAGGAGGAACTATATGCGCTCATACGATATGAACGTTGAGACTGCCGCTGAGTTATCAGCTGTGAACGACATTCTGGCGTCTATCGGTGAACCTCCGGTATCAACGCTGGAAGGTGACGCTAACGCAGATGCAGCGAACGCCCGGCGTATTCTCAACAAGATTAACCGACAGATTCAATCTCGTGGATGGACGtt                                                                                                                                   >  1:507616/1‑301 (MQ=255)`  
                                                                                                                                                                           `gctAACAAAGCCCGAAAGGAAGCTGAGTTGGCTGCTGCCACCGCTGAGCAATAACTAGCATAACCCCTTGGGGCCTCTAAACGGGTCTTGAGGGGTTTTTTGCTGAAAGGAGGAACTATATGCGCTCATGCGATATGAACGTTGAGACTGCCGCTGAGTTATCAGCTGTGAACGACATTCTGGCGTCTATCGGTGAACCTCCGGTATCAACGCTGGAAGGTGACGCTAACGCAGATGCAGCGAACGCTCGGCGTATTCTCAACAAGATTAACCGACAGATTCAATCTCGTGGATGGACGtt                                                                                                                                   >  1:241785/1‑301 (MQ=255)`  
                                                                                                                                                                                      `agacagggAAGCTGAGTGGGCTGCTGCCACCGCTGAGCAATAACTAGCATAACCCCTTGGGGCCTCTAAACGGGTCTTGAGGGGTTTTTTGCTGAAAGGAGGAACTATATGCGCTCATACGATATGAACGTTGAGACTGCCGCTGAGTTATCAGCTGTGAACGACATTCTGGCGTCTATCGGTGAACCTCCGGTATCAACGCTGGAAGGTGACGCTAACGCAGATGCAGCGAACGCTCGGCGTATTCTCAACAAGATTAACCGACAGATTCAATCTCGTGGATGAACGTTCAACATTGAgg                                                                                                                        <  1:172756/295‑1 (MQ=255)`  
                                                                                                                                                                                             `gAAGCTGAGTTGGCTGCTGCCACCGCTGAGCAATAACTAGCATAACCCCTTGGGGCCTCTAAACGGGTCTTGAGGGGTTTTTTGCTGAAAGGAGGAACTATATGCGCTCATGCGATATGAACGTTGAGACTGCCGCTGAGTTATCAGCTGTGAACGACATTCTGGCGTCTATCGGTGAACCTCCGGTATCAACGCTGGAAGGTGACGCTAACGCAGATGTAGCGAACGCTCGGCGTATTCTCAACAAGATTAACCGACAGATTCAATCTCGTGGATGGACGTTCAACAtctgtctcttata                                                                                                                 >  1:493596/1‑289 (MQ=255)`  
                                                                                                                                                                                                     `gTTGGCTGCTGCCACCGCTGAGCAATAACTAGCATAACCCCTTGGGGCCTCTAAACGGGTCTTGAGGGGTTTTTTGCTGAAAGGAGGAACTATATGCGCTCATACGATATGAACGTTGAGACTGCCGCTGAGTTATCAGCTGTGAACGACATTCTGGCGTCTATCGGTGAACCTCCGGTATCAACGCTGGAAGGTGACGCTAACGCAGATGCAGCGAACGCCCGGCGTATTCTCAACAAGATTAACCGACAGATTCAATCTCGTGGCTGGACGTTCAACATTGAGGAAGGCATAACGatac                                                                                                         >  1:758413/1‑301 (MQ=255)`  
                                                                                                                                                                                                        `gagacaggcCACCGCTGAGCAATAACTAGCATAACCCCTTGGGGCCTTTAAACGGGTCTTGAGGGGTTTTTTGCTGAAAGGAGGAACTATATGCGCTCATGCGATATGAACGTTGAGACTGCCGCTGAGTTATCAGCTGTGAACGACATTCTGGCGTCTATCGGTGAACCTCCGGTATCAACGCTGGAAGGTGACGCTAACGCAGATGCAGCGAACGCTCGGCGTATTCTCAACAAGATTAACCGACAGATTCAATCTCGTGGATGGACGTTCAACATTGAGGAAGGCATAACGCtactac                                                                                                      <  1:234531/294‑1 (MQ=255)`  
                                                                                                                                                                                                          `ctgctgCCACCGCTGAGCAATAACTAGCATAACCCCTTGGGGCCTCTAAACGGGTCTTGAGGGGTTTTTTGCTGAAAGGAGGAACTATATGCGCTCATACGATATGAACGTTGAGACTGCCGCTGAGTTATCAGCTGTGAACGACATTCTGGCGTCTATCGGTGAACCTCCGGTATCAACGCTGGAAGGTGACGCTAACGCAGATGCAGCGAACGCTCGGCGTATTCTCAACAAGATTAACCGACAGATTCAATCTCGTGGATGGACGTTCAACATTGAGGAAGGCATAACGCTACTACCt                                                                                                    <  1:184895/301‑1 (MQ=255)`  
                                                                                                                                                                                                              `tgcCACCGCTGAGCAATAACTAGCATAACCCCTTGGGGCCTCTAAACGGGTCTTGAGGGGTTTTTTGCTGAAAGGAGGAACTATATGCGCTCATGCGATATGAACGTTGAGACTGCCGCTGAGTTATCAGCTGTGAACGACATTCTGGCGTCTATCTGTGAACCTCCGGTATCAACGCTGGAAGGTGACGCTAACGCAGATGCAGCGAACGCTCGGCGTATTCTCAACAAGATTAACCGACAGATTCAATCTCGTGTATGGCCGTTCACCATTGAGGACGGCATAACGCTACTACCTGAtt                                                                                                >  1:330497/1‑300 (MQ=255)`  
                                                                                                                                                                                                                 `aagagacaggCAATAACTAGCATAACCCCTTGGGGCCTCTAAACGGGTCTTGAGGGGTTTTTTGCTGAAAGGAGGAACTATATGCGCTCAGGCGATATGAACGTTGAGACTGCCGCTGAGTTATCAGCTGTGAACGACATTCTGGCGTCTATCGGTGAACCTCCGGTATCAACGCTGGAAGGTGACGCTAACGCAGATGCAGCGAACGCTCGGCGTATTCTCAACAAGATTAACCGACAGATTCAATCTCGTGGATGGACGTTCAACATTGAGGAAGGCATAACGCTACTACCTGATGttt                                                                                             <  1:219585/292‑1 (MQ=255)`  
                                                                                                                                                                                                                          `gCAATAACTAGCATAACCCCTTGGGGCCTCTAAACGGGTCTTGAGGGGTTTTTTGCTGAAAGGAGGAACTATATGCGCTCATACGATATGAACGTTGAGACTGCCGCTGAGTTATCAGCTGTGAACGACATTCTGGCGTCTATCGGTGAACCTCCGGTATCAACGCTGGAAGGTGACGCTAACGCAGATGCAGCGAACGCTCGGCGTATTCTCAACAAGATTAACCGACAGATTCAATCTCGTGGATGGACGTTCAACATTGAGGAAGGCATAACGcctggctcttatacacatctccgag                                                                                    >  1:44805/1‑277 (MQ=255)`  
                                                                                                                                                                                                                               `aaCTAGCATAACCCCTTGGGGCCTCTAAACGGGTCTTGAGGGGTTTTTTGCTGAAAGGAGGAACTATATGCGCTCATACGATATGAACGTTGAGACTGCCGCAGAGTTATCAGCTGTGAACGACATTCTGGCGTCTATCGGTGAACCTCCGGTATCAACGCTGGAAGGTGACGCTAACGCAGATGCAGCGAACGCTCGGCGTATTCTCAACAAGATTAACCGACAGATTCAATCTCGTGGATGGACGTTCAACATTGAGGAATGCATAACGCTACTACCTGATGTTTACTCCAACCTGAtt                                                                               >  1:649166/1‑301 (MQ=255)`  
                                                                                                                                                                                                                                    `gCATAACCCCTTGGGGCCTCTAAACGGGTCTTGAGGGGTTTTTTTCTTAAAGGATGAACTATATGCGCTCATACGATATGAACGTTGAGACTGCCGCTGAGTTATCAGCTGTGAACGACATTCTGGCGTCTATCGGTGAACCTCCGGTATCAACGCTGGAAGGTGACGCTACCGCAGATGCAGCGAACGCTCGGCGTATTCTCAACAATATTAACCGACAGATTCAATCTCGTGGATGGACGTTCAACATTTAGGAAGGCATAACGCTACTACCTGATGTTTCCTCCAACCTGATTGtata                                                                          >  1:418139/1‑301 (MQ=255)`  
                                                                                                                                                                                                                                    `gCATAACCCCTTGGGGCCTCTAAACGGGTCTTGAGAGGTTTTTTGCTGAAAGGAGGAACTATATGCGCTCATACGATATGAACGTTGATACTGCCGCTTAGTTATCAGCTGTGAACTACTTTCTGGCATCTATCGGTGAACCTCCGGTATCAACGCTGGAAGGTGACGCTAACGCAGATGCAGCGAACGCTCGGCGTATTCTCAACACGATTACCCGACAGATTCACTCTCGTTAATGGACGTTCAACCTTGAGGAAGGCATAACGCTACTACCTGAGGTCTTCTCCACCCTGTTTCtatt                                                                          >  1:212824/1‑300 (MQ=255)`  
                                                                                                                                                                                                                                     `cATAACCCCTTGGGGCCTCTAAACGGGTCTTGTGGGGTTTTTTGCTGAAAGGAGGAACTATATGCGCTCATACGATATGAACGTTGAGACTGCCGCTGAGTTATCAGCTGTGAACGACATTCTGGCGTCTATCGGTGAACCTCCGGTATCAACGCTGGAAGGTGACGCTAACGCAGATGCAGCGAACGCTCGGCGTATTCTCAACAAGATTAACCGACAGATTCAATCTCGTGGATGGACGTTCAACATTGAGGAAGGCATAACGCTACTACCTGATGTTTACTCCAACCTGATTGTATAc                                                                         <  1:566645/301‑1 (MQ=255)`  
                                                                                                                                                                                                                                     `cATAACCCCTTGGGGCCTCTAAACGGGTCTTGAGGGGTTTTTTGCTGAAAGGAGGAACTATATGCGCTCATACGATATGAACGTTGAGACTGCCGCTGAGTTATCAGCTGTGAACGACATTCTGGCGTCTATCGGTGAACCTCCGGTATCAACGCTGGAAGGTGACGCTAACGCAGATGCAGCGAACGCTCGGCGTATTCTCAACAAGATTAACCGACAGATTCGATCTCGTGGATGGACGTTCAACATTGAGGAAGGCATAACGCTACTACCTGATGTTTACTCCAACCTGATTGTATAc                                                                         <  1:301537/301‑1 (MQ=255)`  
                                                                                                                                                                                                                                     `cATAACCCCTTGGGGCCTCTAAACGGGTCTTGAGGGGTTTTTTGCTGAAAGGAGGAACTATATGCGCTCATACGATATGAACGTTGAGACTGCCGCTGAGTTATCAGCTGTGAACGACATTCTGGCGTCTATCGGTGAACCTCCGGTATCAACGCTGGAAGGTGACGCTAACGCAGATGCAGCGAACGCTCGGCGTATTCTCAACAAGATTAACCGACAGATTCAATCTCGTGGATGGACGTTCAACATTGAGGAAGGCATAACGCTACTACCTGATGTTTACTCCAACCTGATTGTATAc                                                                         <  1:166806/301‑1 (MQ=255)`  
                                                                                                                                                                                                                                     `cATAACCCCGTGGGGCCTCTAAACGGGTCTTGAGGGGTTTTTTGCTGAAAGGAGGAACTATATGCGCTCATGCGATATGAACGTTGAGACTGCCGCTGAGTTATCAGCTGTGAACGACATTCTGTCGTCTATCGGTGAACCTCCGGTATCAACGCTGGAAGGTGACGCTAACGCAGATGCAGCGAACGCTCGGCGTATTCTCAACAAGATTAACCGACAGATTCAATCTCGTGGATGGACGTTCAACATTGAGGAAGGCATAACGCTACTACCTGATGTTTACTCCAACCTGATTGTATAc                                                                         <  1:412442/301‑1 (MQ=255)`  
                                                                                                                                                                                                                                     `agacagcccTTGGGGCCTCTAAACGGGTCTTGAGGGGTTTTTTGCTGAAAGGAGGAACTATATGCGCTCATGCGATATGAACGTTGAGACTGCCGCTGAGTTATCAGCTGTGAACGACATTCTGGCGTCTATCGGTGAACCTCCGGTATCAACGCTGGAAGGTGACGCTAACGCAGATGCAGCGAACGCTCGGCGTATTCTCAACAAGATTAACCGACAGATTCAATCTCGTGGATGGACGTTCAACATTGAGGAAGGCATAACGCTACTACCTGATGTTTACTCCAACCTGATTGTATAc                                                                         <  1:112854/295‑1 (MQ=255)`  
                                                                                                                                                                                                                                     `aaTAACCCCTTGGGGCCTCTAAACGGGTCTTGAGGGGTTTTTTGCTGAAAGGAGGAACTATATGCGCTCATACGATATGAACGTTGAGACTGCCGCTGAGTTATCAGCTGTGAACGACATTCTGGCGTCTATCGGTGAACCTCCGGTATCAACGCTGGAAGGTGACGCTAACGCAGATGCAGCGAACGCTCGGCGTATTCTCAACAAGATTAACCGACAGATTCAATCTCGTGGATGGACGTTCAACATTGAGGAAGGCATAACGCTACTACCTGATGTTTACTCCAACCTGATTGTATAc                                                                         <  1:709767/300‑1 (MQ=255)`  
                                                                                                                                                                                                                                       `ccagcccTTGGGGCCTCTAAACGGGTCTTTAGTGGTTTTTTGCTGAAAGGAGGAACTATATGCGCTCATACGATATGAACGTTGAGACTGCCGCTGAGTTATCAGCTGTGAACGACATTCTGGCGTCTATCGGTGAACCTCCGATATCAACGCTGGAAGGTGACGCTAACGCAGATGCAGCGAACGCTCGGCGTATTCTCAACAAGATTAACCGACAGATTCAATCTCGTGGATGGACGTTCAACATTGAGGAAGGCATAACGCTACTACCTGATGTTTACTCCAACCTGATTGTATACAg                                                                       <  1:522906/297‑1 (MQ=255)`  
                                                                                                                                                                                                                                        `agagacagggggCCTCTAAACGGGTCGTGAGGGGTTTTTTGCTGAAAGGAGGAACTAGATGCGCTCATGCGATATGAACGTTGAGACTGCCGCTGAGTTATCAGCTGTGAACGACATTCTGGCGTCTATCGGTGAACCTCCGGTATCAACTCTGGAAGGTGACGCTAACGCAGATGCAGCGAACGCTCGTCGTATTCTCAACAAGATTAACCGACAGATTCAATCTCGTGGATGGACGTTCAACATTGAGGAAGGCATAACGCTACTACCTGATGTTTACTCCAACCTGATTGTATACAGt                                                                      <  1:659426/293‑1 (MQ=255)`  
                                                                                                                                                                                                                                           `cccTTGGGGCCTCTAAACGGGTCTTGAGGGGTTTTTTGCTGAAAGGAGGAACTATATGCGCTCATGCGATATGAACGTTGAGACTGCCGCTGAGTTATCAGCTGTGAACGACATTCTGGCGTCTATCGGTGAACCTCCGGTATCAACGCGGGAAGGTGACGCTAACGCAGATGCAGCGAACGCTCGGCGTATTCTCCACAAGATTAACCGACAGATTCAATCTCGTGGATGGACGTTCAACATTGAGGAATGCATAACGCTACTACCTGATGTTTACTCCAACCTGATTGTATACAGTgac                                                                   >  1:803868/1‑301 (MQ=255)`  
                                                                                                                                                                                                                                           `cccTTGGGGCCTCTAAACGGGTCTTGAGGGGTTTTTTGCTGAAAGGAGGAACTATATGCGCTCATACGATATGCACGTTGAGACTGCCGCCGAGTTATCAGCTGTGAACGACATTCTGGCGTCTATCGGTGAACCTCCGGTATCAACGCTGGAAGGTGACGCTAACGCAGATGCAGCGAACGCTCGGCGTATTCTCAACAAGATTAACCGACAGATTCAATCTCGTGGATGGACGTTCAACATTTAGACAGGCATCGCGCTACTCCCTCTTGTTTCCTCCAACCTGATTGTATAcctgtct                                                                   >  1:369738/1‑295 (MQ=255)`  
                                                                                                                                                                                                                                                    `ccTCTAAACGGGTCTTGAGGGGTTTTTTGCTGAAAGGAGGAACTATATGCGCTCATGCGATATGAACGTTGAGACTGCCGCTTAGTTATCAGCTGTGAACGACATTCTGGCGTCTATCGGTGAACCTCCGGTATCAACGCTGGAAGGTGACGCTAACGCAGATGCAGCGAACGCTCGGCGTATTCTCAACAAGATTAACCGACAGATTCAATCTCGTGGATGGACGTTCAACATTGAGGAAGGCATAACGCTACTACCTGATGTTTACTCCAACCTGATTGTATAcctgtctcttataaac                                                          >  1:31468/1‑286 (MQ=255)`  
                                                                                                                                                                                                                                                               `gTCTTGAAGGGTTTATTTCTGAAAAGAGGAACTAAAAGCGCTCATACGATATGAACGGTGAGACTGCCACTGAGTTAACAGCTGAGAACGACATTCTGGCGTCTATCGGTGAACCTCCGGTATCAACGCTGGAAGGTGACGCTAACGCAGATGCAGCGAACGCGCGGCGTATTCTCAACAAGACTAACCGACAGATTCAATCTCGTGGATGGACGTTCAACATTGAGGAAGGCATAACGCTACTACCTGATGTTTACTCCAACCTGATTGTATACAGTGACGACTATTTATCCCTAATGTc                                               <  1:390922/301‑1 (MQ=255)`  
                                                                                                                                                                                                                                                                 `cTTGAGGGGTTTTTTGCTGAAAGGAGGAACTATATGCGCTCATACGATATGAACGTTGAGACTGCCGCTGAGTTATCAGCTGTGAACGACATTCTGGCGTCTATCGGTGAACCGCCGGTATCAACGCTGGAAGGTGACGCTAACGCAGATGCAGCGAACGCTCGGCGTATTCTCAACAAGATTAACCGACAGATTCAATCTCGTGGATGGACGTTCAACATTGAGGAAGGCATAACGCTACTACCTGATGTTTACTCCAACCTGATTGTATACAGTGACGACTATTTATCCCTAATGTCTa                                             >  1:515114/1‑301 (MQ=255)`  
                                                                                                                                                                                                                                                                     `aGGGGTTTTTTGCTGAAAGGAGGAACTATATGCGCTCATACGATATGAACGTTGAGACTGCCGCTGAGTTATCAGCTGTGAACGACATTCTGGCGTCTATCGGTGAACCTCCGGTATCAACGCTGGAAGGTGACGCTAACGCAGATGCAGCGAACGCTCGGCGTATTCTCAACAAGATTAACCGACAGATTCAATCTCGTGGATGGACGTTCAACATTGAGGAAGGCATAACGCTACTACCTGATGTTTACTCCAACCTGATTGTATACAGTGACGACTATTTATCCCTAATGTCTACTTc                                         <  1:294250/301‑1 (MQ=255)`  
                                                                                                                                                                                                                                                                     `aGGGGTTTTTTGCTGAAAGGAGGAACTATATGCGCTCATACGATATGAACGTTGAGACTGCCGCTGAGTTATCAGCTGTGAACGACATTCTGGCGTCTATCGGTGAACCTCCGGTATCAACGCTGGAAGGTGACGCTAACGCAGATGCAGCGAACGCTCGGCGTATTCTCAACAAGATTAACCGACAGATTCAATCTCGTGGATGGACGTTCAACATTGAGGAAGGCATAACGCTACTACCTGATGTTTACTCCAACCTGATTGTATACAGTGACGACTATTTATCCCTAATGTCTACTTc                                         <  1:376787/301‑1 (MQ=255)`  
                                                                                                                                                                                                                                                                                   `gAAAGGAGGAACTATATGCGCTCATACGATATGAACGTTGAGACTGCCGCTGAGTTATCAGCTGTGAACGACATTCTGGCGTCTATCGGTGAACCTCCGGTATCAACGCTGGAAGGTGACGCTAACGCAGATGCAGCGAACGCTCGGCGTATTCTCAACAAGATTAACCGACAGATTCAATCTCGTGGATGGACGTTCAACATTGAGGAAGGCATAACGCTACTACCTGATGTTTACTCCAACCTGATTGTATACAGTTACGACTATTTATCCCTAATGTCTACTTCCGGTCAATCCATCt                           >  1:397542/1‑301 (MQ=255)`  
                                                                                                                                                                                                                                                                                     `aaGGAGGAACTATATGCGCTCATGCGATATGAACGTTGAGACTGCCGCTGAGTTATCAGCTGTGAACGACATTCTGGCGTCTATCGGTGAACCTCCGGTATCAACGCTGGAAGGTGACGCTAACGCAGATGCAGCGAACGCTCGGCGTATTCTCAACAAGATTAACCGACAGATTCAATCTCGTGGATGGACGTTCAACATTGAGGAAGGCATAACGCTACTACCTGATGTTTACTCCAACCTGATTGTATACAGTGACGACTATTTATCCCTAATGTCTACTTCCGGTCAATCAATCTAc                         >  1:42532/1‑301 (MQ=255)`  
                                                                                                                                                                                                                                                                                      `aggcggAACTATATGCGCTCATACGATATGAACGTTGAGACTGCCGCTGAGTTATCAGCTGTGAACGACATTCTGGCGTCTATCGGTGAACCTCCGGTATCAACGCTGGAAGGTGACGCTAACGCAGATGCAGCGAACGCTCGGCGTATTCTCAACAAGATTAACCGACAGATTCAATCTCGTGGATGGACGTTCAACATTGAGGAAGGCATAACGCTACTACCTGATGTTTACTCCAACCTGATTGTATACAGTGACGACTATTTATCCCTAATGTCTACTTCCGGTCAATCCATCTACg                        >  1:608069/1‑301 (MQ=255)`  
                                                                                                                                                                                                                                                                                       `ggaggaACTATATGCGCTCATACGATATGAACGTTGAGACTGCCGCTGAGTTATCAGCTGTGAACGACATTCTGGCGTCTATCGGTGAACCTCCGGTATCAACGCTGGAAGGTGACGCTAACGCAGATGCAGCGAACGCTCGGCGTATTCTCAACAAGATTAACCGACAGATTCAATCTCGTGGATGGACGTTCAACATTGAGGAAGGCATAACGCTACTACCTGATGTTTACTCCAACCTGATTGTATACAGTGACGACTATTTATCCCtgtctcttatacccctctccgagcccacgag                       >  1:682261/1‑271 (MQ=255)`  
                                                                                                                                                                                                                                                                                        `gaggaACTATATGCGCTCATACGATATGAACGTTGAGACTGCCGCTGAGTTATCAGCTGTGAACGACATTCTGGCGTCTATCGGTGAACCTCCGGTATCAACGCTGGAAGGTGACGCTAACGCAGATGCAGCGAACGCTCGGCGTATTCTCAACAAGATTAACCGACAGATTCAATCTCGTGGATGGACGTTCAACATTGAGGAAGGCATAACGCTACTACCTGATGTTTACTCCAACCTGATTGTATACAGTGACGACTATTTATCCCTAATGTCTAcctgtctcttatacacatctctg                      >  1:100002/1‑279 (MQ=255)`  
                                                                                                                                                                                                                                                                                          `ggaACTATATGCGCTCATGCGATATGAACGTTGAGACTGCCGCTGAGTTATCAGCTGTGAACGACATTCTGTCGTCTATCGGTGAACCTCCGGTATCAACGCTGGAAGGTGACGCTAACGCAGATGCAGCGAACGCTCGGCGTATTCTCAACAAGATTAACCGACAGATTCAATCTCGTGGATGGACGTTCAACATTGAGGAAGGCATAACGCTACTACCTGATGTTTACTCCAACCTGATTGTATACAGTGACGACTATTTATCCCTAATGTCTACTTCCGGTCAATCCATCTACGTTaa                    >  1:134818/1‑301 (MQ=255)`  
                                                                                                                                                                                                                                                                                           `aaaCTATATGCGCTCATACGATATGAACGTTGAGACTGCCGCTGAGTTATCAGCTGTGAACGACATTCTGGCGTCTATCGGTGAACCTCCGGTATCAACGCTGGAAGGTGACGCTAACGCAGATGCAGCGAACGCTCGGCGTATTCTCAACAAGATTAACCGACAGATTCAATCTCGTGGATGGACGTTCAACATTGAGGAAGGCATAACGCTACTACCTGATGTTTACTCCAACCTGATTGTATACAGTGACGACTATTTATCCCTAATGTCTACTTCCGGTCAATCCATCTACGTTAAc                   <  1:59256/300‑1 (MQ=255)`  
                                                                                                                                                                                                                                                                                            `ctatactcgcgcTCATACGATATGAACTTTGAGACTGCCGCTGAGTTATCAGCTTTGAACGACATTCTGGCGTCTATCGGTGAACCTCCGGTATCAACGCTGGAAGGTGACGCTAACGCAGATGCAGCGAACGCTCGGCGTATTCTCAACAAGATTAACCGACAGATTCAATCTCGTGGATGGACGTTCAACATTGAGGAAGGCATAACGCTACTACCTGATGTTTACTCCAACCTGATTGTATACAGTGACGACTATTTATCCCTAATGTCTACTTCCGGTCAATCCATCTACGTTAAcc                  <  1:828927/293‑1 (MQ=255)`  
                                                                                                                                                                                                                                                                                            `aaCTATATGCGCTCATACGATATGAACGTTGAGACTGCCGCTGAGTTATCAGCTGTGAACGACATTCTGGCGTCTATCGGTGAACCTCCGGTATCAACGCTGGAAGGTGACGCTAACGCAGATGCAGCGAACGCTCGGCGTATTCTCAACAAGATTAACCGACAGATTCAATCTCGTGGATGGACGTTCAACATTGAGGAAGGCATAACGCTACTACCTGATGTTTACTCCAACCTGATTGTATACAGTGACGACTATTTATCCCTAATGTCTACTTCCGGTCAATCCATCTACGTTAAcc                  <  1:20681/301‑1 (MQ=255)`  
                                                                                                                                                                                                                                                                                               `tataTGCGCTCATGCGTTATGAACGTTTAGACTGCCGCTGAGTTATCAGCTGTCAACGACATTCTGTCGACTATCGGTGAACCTCCGTTTTCAACGCTGGAAGTTTACGCTAACGCAGATGCCGCGAACGCTTGGTGTATTCTCAACTAGATTAACCGACAGATTCAATCTCGTGGATGGACGCTCAACCTTTAGGAAGGCATAATGCCACTACCTGCTGTTTTCTTCATCCTGTTTGTATACAGTGACGACTATTTATTCTTAATATTTACCTCCGTGCTATCCATCTTCGTTACCCCAg               >  1:279723/1‑301 (MQ=255)`  
                                                                                                                                                                                                                                                                                                `atatGCGATCATACGATATGAACGTTGAGACTGCCGCTGAGTTATCAGCTGTGAACGACATTCTGGCGTCTATCGGTGAACCTCCGGTATCAACGCTGGAAGGTGACGCTAACGCAGATGCAGCGAACGCTCGGCGTATTCTCAACAAGATTAACCGACAGATTCAATCTCGTGGATGGACGTTCAACATTGAGGAAGGCATAACGCTACTACCTGATGTTTACTCCAACCTGATTGTATACAGTGACGACTATTTATCCCTAATGTCTACTTCCGGTCAATCCCTCTACGTTAACCgctg              >  1:600305/1‑298 (MQ=255)`  
                                                                                                                                                                                                                                                                                                          `aTACGATATGAACGTTGAGACTGCCGCTGAGTTATCAGCTGTGAACGACATTCTGGCGTCTATCGGTGAACCTCCGGTATCAACGCTGGAAGGTGACGCTAACGCAGATGCAGCGAACGCTCGGCGTATTCTCAACAAGATTAACCGACAGATTCAATCTCGTGGATGGACGTTCAACATTGAGGAAGGCATAACGCTACTACCTGATGTTTACTCCAACCTGATTGTATACAGTGACGACTATTTATCCCTAATGTCTACTTCCGGTCAATCCATCTACGTTAACCGAGGTGGCTAtgtg    <  1:344690/301‑1 (MQ=255)`  
                                                                                                                                                                                                                                                                                                          `aTACGAGAGTAACGTGGAGACTGCCGCTGATTTATCAGCTGTGAACGACATTCTGTCGTCGATCGGTGAACCTCCGGTATCAACGCTGGAAGGTGACGCTAACGCAGATGCAGCGAACGCTCGGCGTATTCTCAACAAGATTAACCGACAGATTCAATCTCGTTGATGGACGTTCAACATTGAGGAAGGCATAACGCTACTACCTGATGTTTACTCCAACCTGATTGTATACAGTGACGACTATTTATCCCTAATGTCTACTTCCGGTCAATCCATCTACGTTAACCGAGGTGGCTAtgtg    <  1:501521/301‑1 (MQ=255)`  
                                                                                                                                                                                                                                                                                                           `aaCGATAAGAACGTGGAGGCTGCCGGTGAATTATAAGCTGTGAACGACATTCTGGCGTCTATCGGTGAACCTCCGGTATCGACGCTGGAAGGTGACGCTAACGCAGATGCAGCGAACGCTCGGCGTATTATCAACAAGATTAACCGACAGATTCAATCTCGTGGATGGACGTTCAACATTGAGGAAGGCATAACGCTACTACCTGATGTTTACTCCAACCTGATTGTATACAGTGACGACTATTTATCCCTAATGTCTACTTCCGGTCAATCCATCTACGTTAACCGAGGTGGCTATgtgt   <  1:766365/300‑1 (MQ=255)`  
                                                                                                                                                                                                                                                                                                            `aCGATATGAACGTTGAGACTGCCGCTGAGTTATCAGCTGTGAACGACATTCTGGCGTCTATCGGTGAACCTCCGGTATCAACGCTGGAAGGTGACGCTAACGCAGATGCAGCGAACGCTCGGCGTATTCTCAACAAGATTAACCGACAGATTCAATCTCGTGGATGGACGTTCAACATTGAGGAAGGCATAACGCTACTACCTGATGTTTACTCCAACCTGATTGTATACAGTGACGACTATTTATCCCTAATGTCTACTTCCGGTCAATCCATCTACGTTAACCGAGGTGGCTATGTGTa  <  1:69614/301‑1 (MQ=255)`  

                                                                                                                                                                                                                                                                                                            `|`                                                                                                                                                                                                                                                                                                              
`GTACGCAATGGGCCACGGTGGTCTTCGCCCAGAAGCTGCTGGTGCAGTGGTTTTCAAAGTGGAGTAATGCTGGGGGTGGCCTCAACGGTCGCTGCTAGTCCCGAAGAGGCGAGTGTTACTTCAACAGAAGAAACCTTAACGCCAGCACAGGAGGCCGCACGCACCCGCGCTGCTAACAAAGCCCGAAAGGAAGCTGAGTTGGCTGCTGCCACCGCTGAGCAATAACTAGCATAACCCCTTGGGGCCTCTAAACGGGTCTTGAGGGGTTTTTTGCTGAAAGGAGGAACTATATGCGCTCATACGATATGAACGTTGAGACTGCCGCTGAGTTATCAGCTGTGAACGACATTCTGGCGTCTATCGGTGAACCTCCGGTATCAACGCTGGAAGGTGACGCTAACGCAGATGCAGCGAACGCTCGGCGTATTCTCAACAAGATTAACCGACAGATTCAATCTCGTGGATGGACGTTCAACATTGAGGAAGGCATAACGCTACTACCTGATGTTTACTCCAACCTGATTGTATACAGTGACGACTATTTATCCCTAATGTCTACTTCCGGTCAATCCATCTACGTTAACCGAGGTGGCTATGTGTA  >  NC_001604.1/23938‑24538`

  

Alignment Legend

Aligned base mismatch/match (shaded by quality score): `ATCG/ATCG` `< 3 ≤` `ATCG/ATCG` `< 10 ≤` `ATCG/ATCG` `< 23 ≤` `ATCG/ATCG` `< 35 ≤` `ATCG/ATCG` `< 38 ≤` `ATCG/ATCG`

Unaligned base: `atcg`    Masked matching base: `atcg`    Alignment gap: `‑`    Deleted base: `‑`
